# Material decomposition approaches for monosodium urate (MSU) quantification in gouty arthritis: a (bio)phantom study

**DOI:** 10.1186/s41747-024-00528-z

**Published:** 2024-11-08

**Authors:** Torsten Diekhoff, Sydney Alexandra Schmolke, Karim Khayata, Jürgen Mews, Maximilian Kotlyarov

**Affiliations:** 1 Department of Radiology, Charité—Universitätsmedizin Berlin, Campus Mitte, Humboldt-Universität zu Berlin, Freie Universität Berlin, Berlin, Germany; 2 Canon Medical Systems Europe BV, Global Research & Development Center, Amstelveen, The Netherlands

**Keywords:** Arthritis (gouty), Image processing (computer-assisted), Phantoms (imaging), Tomography (x-ray computed), Uric acid

## Abstract

**Background:**

Dual-energy computed tomography (DECT) is a noninvasive diagnostic tool for gouty arthritis. This study aimed to compare two postprocessing techniques for monosodium urate (MSU) detection: conventional two-material decomposition and material map-based decomposition.

**Methods:**

A raster phantom and an *ex vivo* biophantom, embedded with four different MSU concentrations, were scanned in two high-end CT scanners. Scanner 1 used the conventional postprocessing method while scanner 2 employed the material map approach. Volumetric analysis was performed to determine MSU detection, and image quality parameters, such as signal-to-noise ratio (SNR) and contrast-to-noise ratio (CNR), were computed.

**Results:**

The material map-based method demonstrated superior MSU detection. Specifically, scanner 2 yielded total MSU volumes of 5.29 ± 0.28 mL and 4.52 ± 0.29 mL (mean ± standard deviation) in the raster and biophantom, respectively, *versus* 2.35 ± 0.23 mL and 1.15 ± 0.17 mL for scanner 1. Radiation dose correlated positively with detection for the conventional scanner, while there was no such correlation for the material map-based decomposition method in the biophantom. Despite its higher detection rate, material map-based decomposition was inferior in terms of SNR, CNR, and artifacts.

**Conclusion:**

While material map-based decomposition resulted in superior MSU detection, it is limited by challenges such as increased artifacts. Our findings highlight the potential of this method for gout diagnosis while underscoring the need for further research to enhance its clinical reliability.

**Relevance statement:**

Advanced postprocessing such as material-map-based two-material decomposition might improve the sensitivity for gouty arthritis in clinical practice, thus, allowing for lower radiation doses or better sensitivity for gouty tophi.

**Key Points:**

- Dual-energy CT showed limited sensitivity for tophi with low MSU concentrations.
- Materiel-map-based decomposition increased sensitivity compared to conventional two-material decomposition.
- The advantages of material-map-based decomposition outweigh lower image quality and increased artifact load.

**Graphical Abstract:**

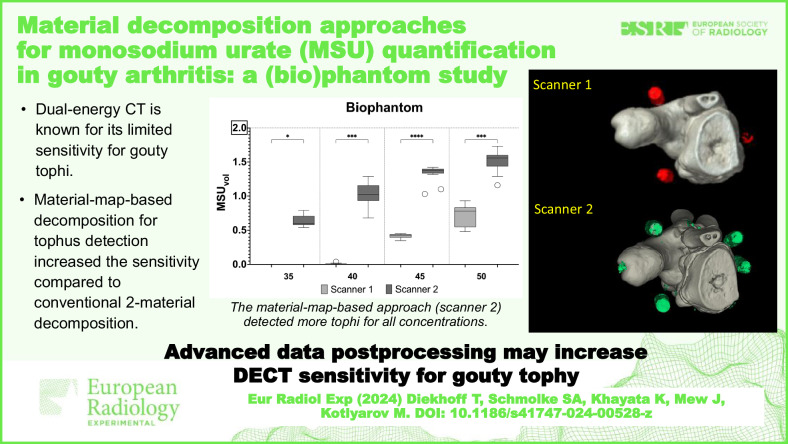

## Background

Dual-energy computed tomography (DECT) has gained value as a noninvasive diagnostic tool for gouty arthritis, enabling not only the identification but also the quantification of monosodium uric acid (MSU) depositions and their response to therapy [1, 2]. However, inconsistencies exist regarding the DECT sensitivity for MSU. Although the majority of studies suggest an approximately 90% sensitivity in gouty arthritis patients, some authors argue that sensitivity declines in early disease and in tophi with relatively low MSU concentration [3].

Generally, a two-material decomposition algorithm differentiates materials with higher HUinto calcium (bone) and MSU based on their respective dual-energy gradient [4]. This gradient, intrinsically linked to the effective atomic number (*Z*_eff_), reflects the HU change resulting when x-ray energy, or tube voltage, is varied. The sensitivity of this technique hinges on a threshold to distinguish MSU from soft tissues in tendons, ligaments, and muscles, which can show similar DE characteristics [5]. Therefore, for accurate MSU detection, the threshold must exclude all physiological, and anatomical structures and is usually set at approximately 150 HU [6]. However, this process omits less dense MSU depositions, thereby degrading sensitivity [7].

**Fig. 1:**
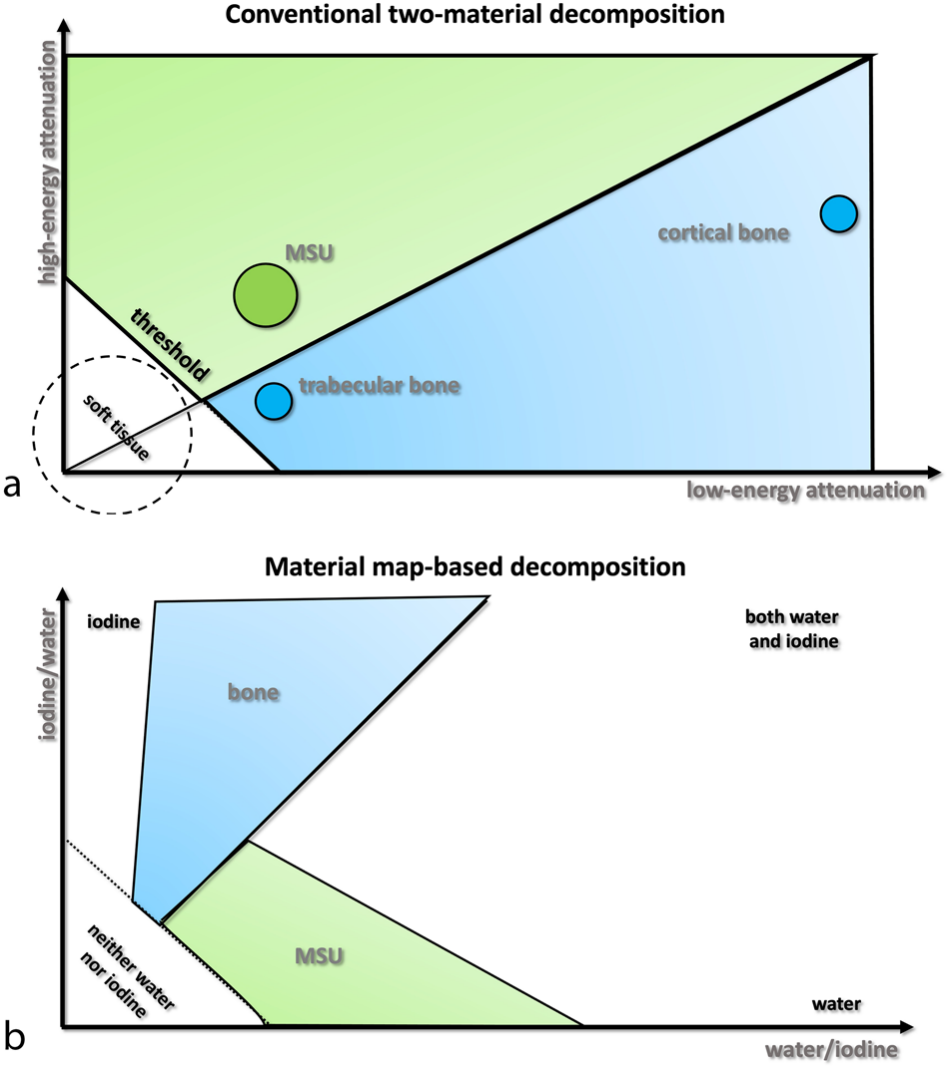
Diagrams illustrating two-material decomposition (**a**) and material map-based decomposition (**b**). MSU, Monosodium uric acid

An alternative technique involves decomposition based on material maps. This method assigns each voxel a probability of containing one of two materials, such as iodine and water, potentially enhancing the differentiation between MSU and bone or soft tissues [8]. Comparison of the two material maps (iodine/water and water/iodine) allows more accurate characterization and differentiation of materials, as they are found in different regions on the graph (Fig. 1).

This study aims to compare the sensitivity of these two postprocessing techniques for MSU detection in a phantom setting.

## Methods

Since this is a phantom study, approval from the institutional review board was not required.

### Phantom model

A raster phantom and an *ex vivo* biophantom were fabricated following an established protocol [5]. Four MSU concentrations (35%, 40%, 45%, and 50%) suspended in ultrasound gel were prepared and inserted into 2 mL syringes. The scanning was conducted within 6 h after manufacturing the suspensions, ensuring the homogenous distribution of MSU within the syringe was retained. For the raster phantom, these four samples and a pure ultrasound gel for negative control were arranged within a plastic container, and subsequently filled with water. For the biophantom, a fresh pig hindleg from the slaughterhouse was used, in which five pockets were bluntly prepared around the joint soft tissues to accommodate the syringes before scanning.

### CT protocol and postprocessing

The phantoms were scanned in two high-end 320-row volume detector CT scanners: Aquilion One Vision Edition (scanner 1, Canon Medical Systems Corporation, Shimoishigami, Otawara-shi, Tochigi, Japan), installed in 2013, and Aquilion One Prism (scanner 2, Canon Medical Systems Corporation), installed in 2021. All scans were performed in volume mode, without table movement, and with full 16-cm *z*-axis coverage. All primary reconstructions were performed with a slice thickness of 0.5 mm and an in-plane resolution of 0.468 mm × 0.468 mm.

#### Conventional material decomposition

The first scanner facilitated dual-energy acquisition through the rotate-rotate method, comprising two volume scans with 135 kVp and 80 kVp. Scans were acquired with an ascending order of tube current-time products: 2.75/16.5 mAs, 4.125/24.75 mAs, 5.5/30.25 mAs, 8.25/46.75 mAs, 11/63.25 mAs, 13.75/79.75 mAs, 19.25/110 mAs, 24.75/140.25 mAs, 30.25/173.25 mAs, and 38.5/220 mAs for the 135 kVp and 80 kVp acquisition, respectively. Images were reconstructed using adaptive iterative dose reduction (AIDR-3D) with a medium soft-tissue kernel without beam-hardening compensation and postprocessed with the traditional two-material decomposition algorithm on the CT console (dual-energy composition analysis, version 6.0, Canon Medical Systems Corporation).

#### Material map-based postprocessing

The second scanner employed a rapid kVp-switching method, altering the tube voltage from 80 kVp to 135 kVp several times within a single acquisition without changing the tube current. Given a rotation time of 0.275 s, the following tube currents were applied: 350 mA, 400 mA, 450 mA, 500 mA, 550 mA, and 600 mA. For a rotation time of 0.5 s, additional acquisitions with 230 mA, 250 mA, 270 mA, and 300 mA were possible. This protocol resulted in a total of 16 distinct acquisitions with scanner 2. Images were reconstructed using spectral advanced intelligent clear-IQ engine (spectral-AICE) and further postprocessed using the dual-energy composition analysis module of a stand-alone Vitrea solution (version 7.14.2.227, Vital Images, Canon Medical Systems Corporation), employing the material map method for gout detection. This technique uses the iodine/water image pairs.

### Measurements

Volumetric analysis was performed to calculate the total MSU volume and the volume detected in each syringe. The artifact volume was determined by subtracting the true-positive volume within the syringes from the total MSU volume. We selected regions of interest within the 50% MSU syringe, water (for the raster phantom) or muscle (for the biophantom), and the surrounding air to compute image quality parameters, namely, signal-to-noise ratio (SNR) and contrast-to-noise ratio (CNR). The signal was defined as the Hounsfield units measured within the syringe, the contrast as its difference compared to water or soft tissues, respectively, and the noise as the standard deviation measured within the air.

### Statistics

Statistical analysis was performed using Prism (version 10, GraphPad, La Jolla, CA, USA). The volumes of the different MSU concentrations were compared using a Kruskal–Wallis test with Dunn’s correction to account for multiple comparisons. We used the Mann–Whitney *U* method to compare artifact volumes and SNR and CNR. A corrected *p*-value of less than 0.05 was considered to indicate statistical significance.

### Language editing

ChatGPT version 4o was used for language editing.

## Results

**Fig. 2:**
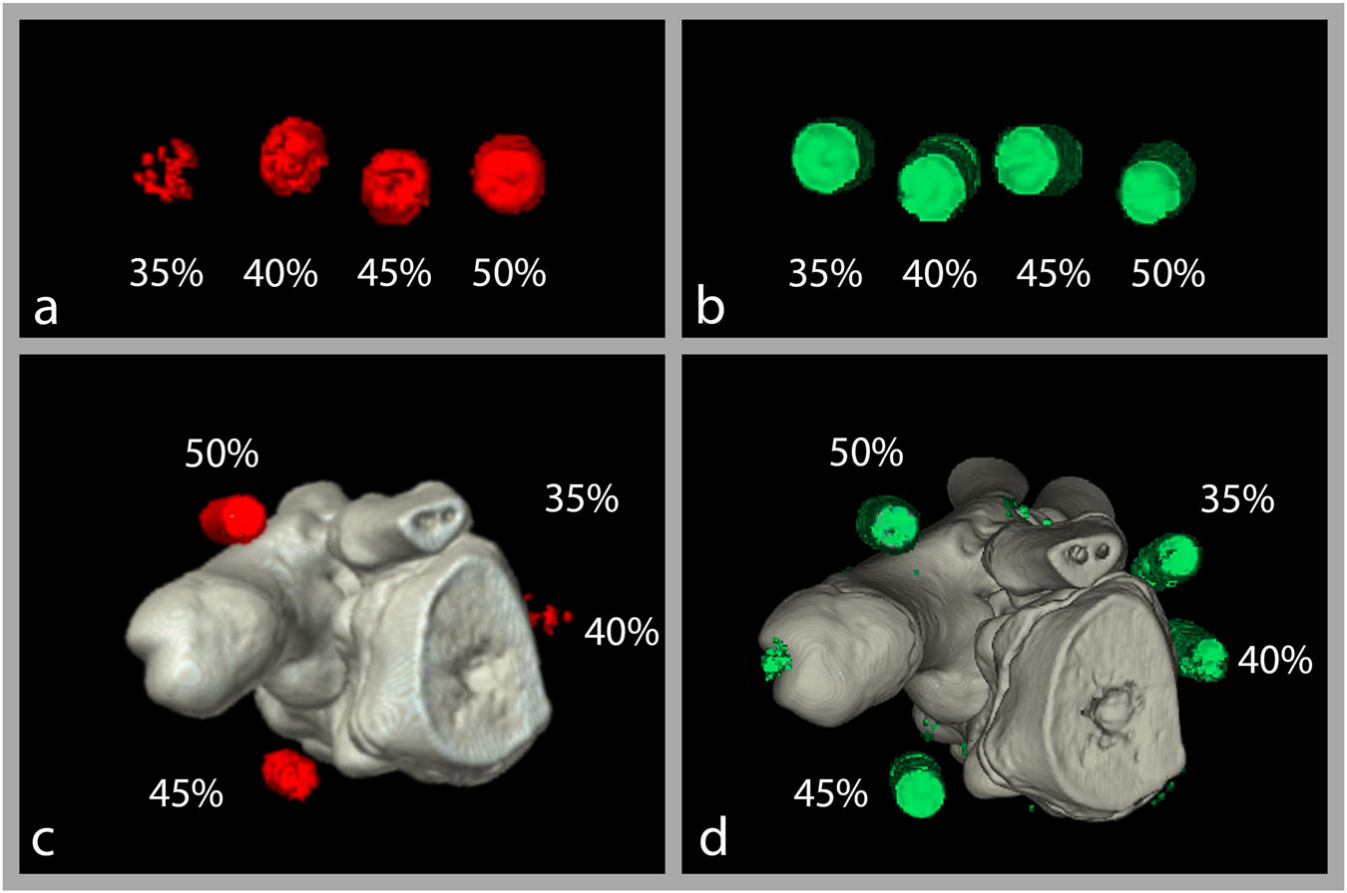
Three-dimensional MSU map of the raster phantom generated using two-material decomposition (**a**) and material map-based decomposition (**b**). Three-dimensional MSU map of the biophantom generated using two-material decomposition (**c**) and material map-based decomposition (**d**). The percentage numbers indicate the MSU concentration of a detected syringe in a phantom. Note the artifacts near the calcaneus in (**d**)

We compared ten acquisitions on scanner 1 and sixteen on scanner 2. The dose-length product ranged from 16.1 mGy × cm to 218.2 mGy × cm (scanner 1), corresponding to an estimated effective dose of 0.006 mSv to 0.087 mSv, using a conversion factor of 0.0004 for upper extremities [9]. For scanner 2, the dose-length product ranged from 72.3 mGy × cm to 217.5 mGy × cm with an estimated effective dose of 0.029 mSv to 0.087 mSv. Example images are shown in Fig. 2.

### Volumetric analysis

**Fig. 3:**
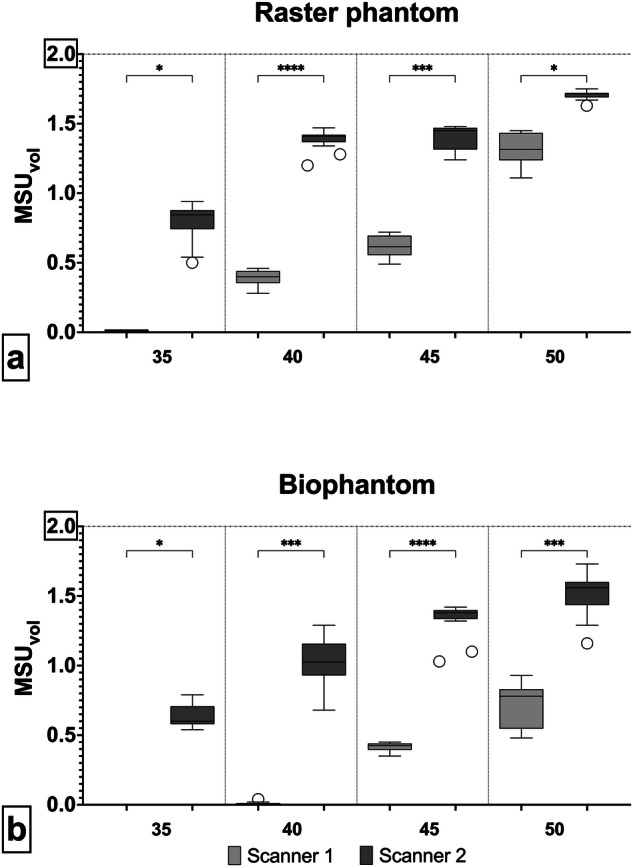
Comparison of detected volumes on scanner 1 and scanner 2 for different MSU concentrations in the raster phantom (**a**) and in the biophantom (**b**). Scanner 2 detected significantly higher MSU volumes at all four concentrations. The ground truth and maximum detectable volume for every concentration is 2 mL (horizontal line)

Four suspensions with different concentrations, each with a volume of 2 mL, were manufactured, resulting in a total of 8 mL of MSU suspension. We detected significantly more MSU on scanner 2 compared to scanner 1, with total volumes of 5.29 ± 0.28 mL *versus* 2.35 ± 0.23 mL for the raster phantom (*p* < 0.001), and 4.52 ± 0.29 mL *versus* 1.15 ± 0.17 mL for the biophantom (*p* < 0.001), respectively. Figure 3 displays the results for the different MSU concentrations.

**Fig. 4:**
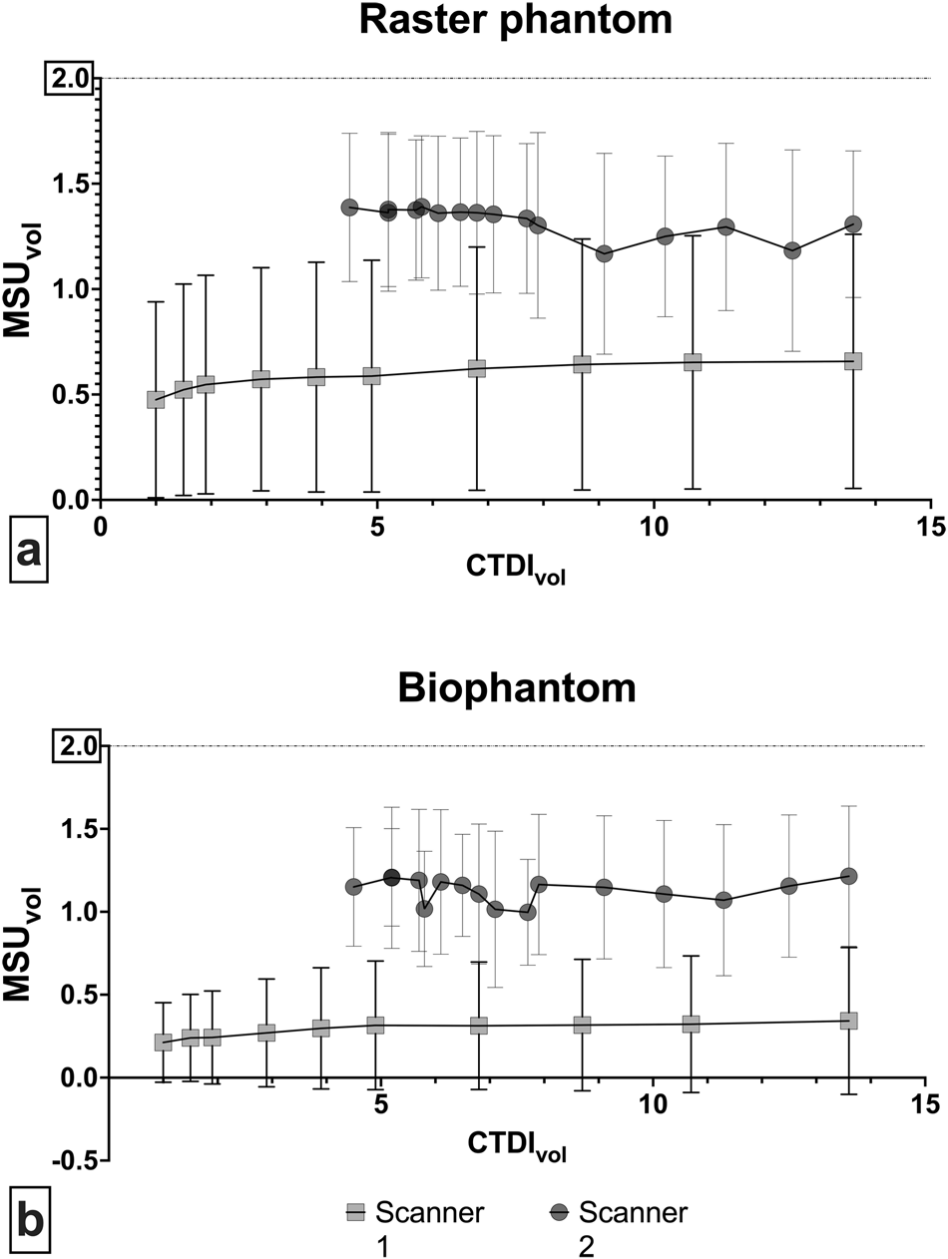
Plot of detected MSU volume (MSU_vol_) against the CT dose index volume (CTDI_vol_) for the raster (**a**) and the biophantom (**b**). The graph displays the mean for all four concentrations. The maximum detectable concentration per syringe is 2 mL (horizontal line). While detection with conventional postprocessing (scanner 1) positively correlates with the radiation dose, no such relationship is observed for scanner 2

There was a significant correlation between the computed tomography (CT) dose index and the detected volume for scanner 1 in both the raster phantom (*r* = 0.91, *p* < 0.001) and the biophantom (*r* = 0.88, *p* = 0.001). However, for scanner 2, we observed a negative correlation in the raster phantom (*r* = -0.73, *p* = 0.001) and no correlation in the biophantom (*r* < 0.001, *p* = 0.99). The corresponding graphs are shown in Fig. 4. Dose effectiveness as calculated by the detected MSU volume per dose unit is shown in Supplementary Fig. S1.

**Fig. 5:**
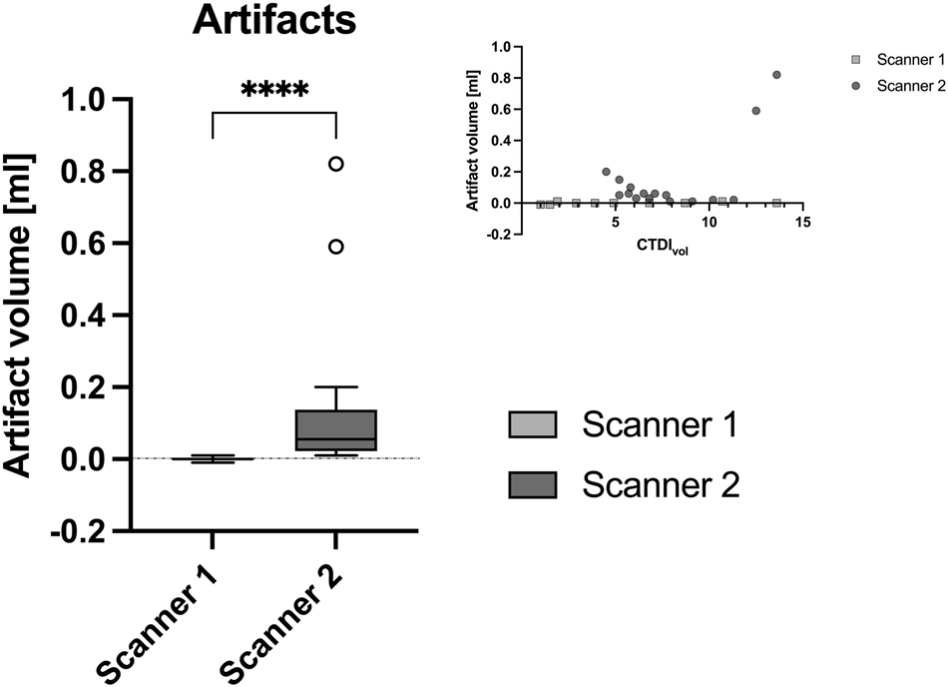
Comparison of artifact volumes for the two scanners in the biophantom. The graph represents the false-positive volumes for each of the two scanners in relation to the radiation exposure (top right)

**Fig. 6:**
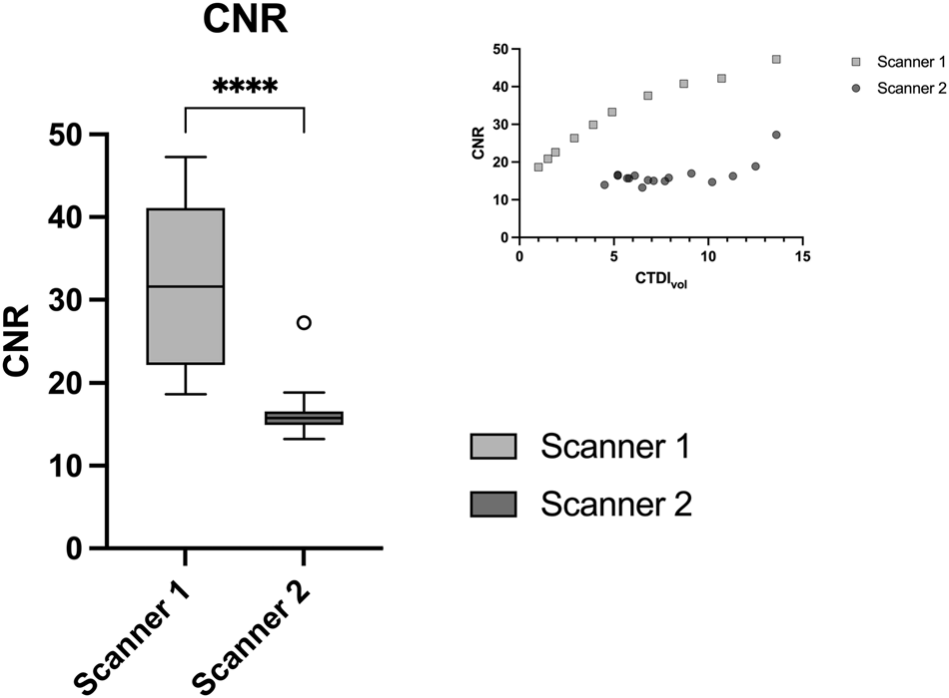
CNR for the two scanners. Spectral acquisition with scanner 2 shows lower CNR values compared with scanner 1. However, for both scanners, CNR gradually improves with increasing radiation exposure

Figure 5 illustrates the artifact volumes for both scanners. Interestingly, scanner 2 showed more artifacts, *i.e*., beam hardening near the bone surface. These artifacts were most pronounced in the scans with very high radiation exposure. Notably, these artifacts did not correlate with image quality (see CNR values in Fig. 6). The SNR values were 53.6 ± 16.3 for scanner 1 and 17.5 ± 3.2 for scanner 2, respectively (*p* < 0.001).

## Discussion

Our study provides a comparative evaluation of two DECT postprocessing techniques: conventional two-material decomposition (scanner 1) and novel material map-based decomposition (scanner 2). Overall, our results showed that the material map-based approach, despite certain drawbacks, consistently outperformed the traditional decomposition technique in detecting the volume of MSU crystals, especially under analogous radiation exposure conditions. The material map-based decomposition technique detected significantly larger MSU volumes in both the raster phantom and the *ex vivo* biophantom. This result could be attributable to the enhanced material differentiation capability of the material map technique, which improves the differentiation of MSU, bone, and soft tissues by assigning a probability value to each voxel for containing a specific material (such as iodine or water).

Furthermore, an interesting pattern emerged regarding the relationship between the CT dose index volume (CTDI_vol_) and the detected MSU volumes. For scanner 1 using conventional decomposition, there was a strong positive correlation between the radiation dose and the detected volume, indicating that a higher radiation dose results in better detection. Conversely, scanner 2 using the material map-based decomposition technique showed no such correlation in the biophantom model, suggesting that its superior detection capability is independent of the applied radiation dose, at least within the limits of our study. This feature could pave the way for further dose-reduction strategies without compromising diagnostic performance.

However, it is noteworthy that, while the material map-based decomposition method provided superior detection rates, it did encounter a few challenges. Images acquired with scanner 2 showed more artifacts, particularly beam hardening near the bone surface, and more false-positive detections within soft tissues. This may lead to overcalling gouty arthritis in clinical practice and could be attributable to the inherent characteristics of the rapid kVp-switching method employed in scanner 2, which uses fewer projections for the acquisition of each basic dataset and does not permit independent adjustment of tube current for each tube voltage. In terms of objective image quality parameters, scanner 2 showed inferior SNR and CNR values compared to scanner 1. While these findings could suggest a potential drawback in image quality, it is important to note that the superior MSU detection achieved using material map-based decomposition far outweighs these minor limitations, making it an effective choice for gout diagnosis.

Previous research has highlighted the importance of reducing image noise, which can be accomplished by applying iterative reconstruction, in enhancing the detection of tophi, especially when the detection threshold is tailored to match the respective noise level. Similarly, tube current settings for dual-energy scans have been identified to significantly influence the detection rate and radiation exposure [10]. The efficacy of DECT in detecting MSU crystals is known to be significantly influenced by the concentration of MSU within a tophus [11]. Consequently, the sensitivity of DECT is limited in early disease or when the tophus is either liquified, calcified, or enhanced with a contrast medium [3, 12, 13]. Therefore, while DECT proves advantageous in established gout cases characterized by a high concentration of MSU crystals, it might be less effective in early diagnosis or different stages of tophus transformation. In particular, DECT has demonstrated potential for differentiating crystal depositions in a phantom model [11, 14] and that characterization of these depositions might be possible with comparably low radiation exposure [15]. However, these findings need to be interpreted cautiously, as there remains a degree of skepticism in their clinical applicability [16].

While experience with photon-counting detector CT for crystal arthropathies is still limited [17], this emerging technology, which allows the precise counting of individual photons, promises significant advancements in CT imaging by enhancing spatial resolution, reducing image noise, and improving energy resolution for more accurate material differentiation. It is plausible that the benefits of this technology, coupled with optimized postprocessing, will contribute to a better diagnosis in the field of crystal arthropathy diagnosis in the near future.

The challenge lies in translating our promising phantom model results into clinically reliable tools. The complexities of human anatomy, varying stages and presentations of the disease, and the presence of other comorbidities can significantly impact the diagnostic performance of these techniques in real-world settings.

As for the limitations of our study, we analyzed a restricted set of MSU concentrations based on our previous studies. Consequently, we were unable to definitively establish the ultimate detection threshold offered by the novel, material map-based decomposition approach of scanner 2. Further research is needed to clarify this aspect. Although carefully considered, the position of the porcine limb might have introduced bias. It is also important to consider that, while both scanners used DECT technology, they differed in terms of postprocessing methods, as well as scanning and reconstruction techniques. This divergence limits the ultimate comparability of the two CT scanners and could have introduced confounders into our analysis [18]. Regrettably, the proprietary software used by each scanner is incompatible with the source data of the other scanner, which further hinders a more direct comparison. Nevertheless, we are confident that the major impact of our analysis is predominantly based on the differential effect of the reconstruction software used. This confidence is bolstered by the observation that the input data appeared to be of slightly lower quality for scanner 2, which performed better in our experiments despite this apparent disadvantage. However, given the significant differences in scanning and reconstruction techniques, a true comparison of one material decomposition technique to the other is not valid. With the two different phantom settings, we aimed to ascertain the performance of the two decomposition methods unrelated (raster phantom) and surrounded by other tissues like tendons and bone (biophantom). However, a dedicated analysis in settings with pure surrounding soft tissues or bone in a more standardized set-up or with rotation of the biophantom was not performed. The orientation of the syringes in the CT scanner might have introduced bias. Reproducibility may be impeded by anatomical variations in the porcine leg and the requisite manufacturing of new MSU suspensions.

In light of these considerations, our findings provide a critical understanding of the comparative performance of different DECT postprocessing techniques, with implications for enhancing diagnostic accuracy in gout and other crystal arthropathies. However, they also underline the need for more extensive research and clinical trials to translate these promising techniques into practical, clinically reliable diagnostic tools. This includes more studies on emerging technologies in the field of CT imaging and the development of optimized postprocessing strategies that can improve detection, even in early or complex stages of the disease.

## Supplementary information

**Additional file 1:** **Fig. S1:** Comparison of both scanners, the detected volume, and the radiation exposure.

## Data Availability

Source data are available from the authors upon reasonable request.

## Abbreviations

CNR: Contrast-to-noise ratio
CT: Computed tomography
DECT: Dual-energy computed tomography
MSU: Monosodium uric acid
SNR: Signal-to-noise ratio
