# Supplementary material for: Material decomposition approaches for monosodium urate (MSU) quantification in gouty arthritis: a (bio)phantom study
Source: Eur Radiol Exp. 2024 Nov 8;8:127. doi: 10.1186/s41747-024-00528-z (PMC11549270; doi:10.1186/s41747-024-00528-z)
Supplement: Supplementary file 1 — Additional file 1: Fig. S1: Comparison of both scanners, the detected volume, and the radiation exposure. [file 41747_2024_528_MOESM1_ESM.pdf]

Material decomposition approaches for monosodium urate (MSU) quantification in gouty arthritis: a (bio)phantom study

ELECTRONIC SUPPLEMENTARY MATERIAL

Supplement 1: Dose effectiveness

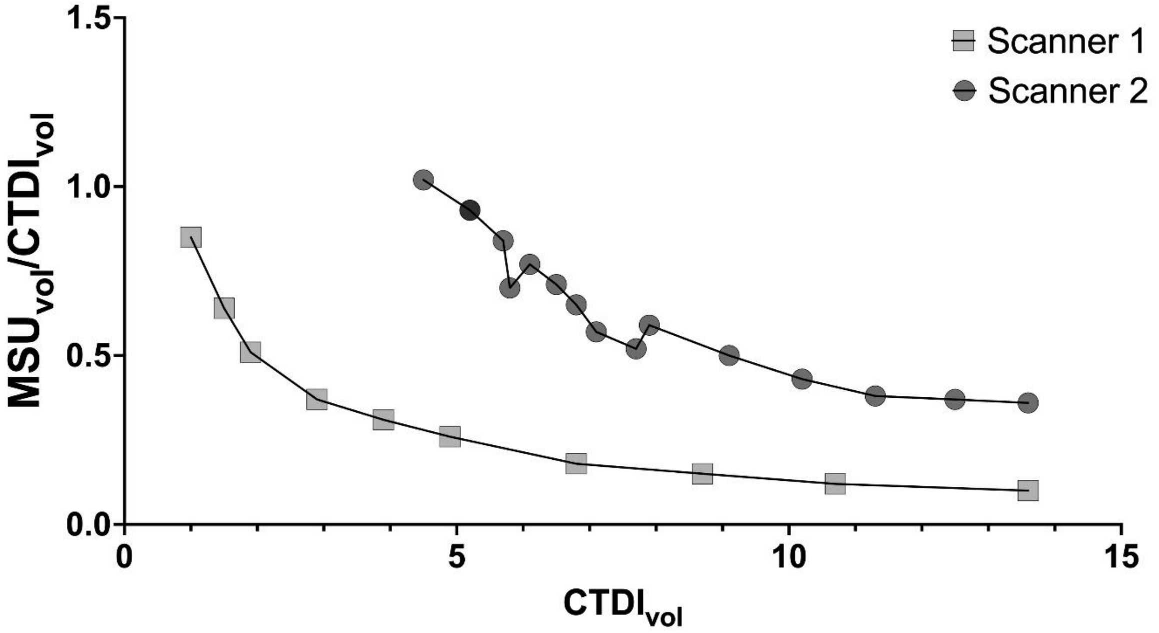

Fig. S1: Comparison of both scanners, the detected volume and the radiation exposure. *MSU_{vol}* Volume of monosodium uric acid given in mL, *CTDIvol* Volumetric computed tomography dose index in mGy.
